# Publisher Correction: Interplay between hypoxia and androgen controls a metabolic switch conferring resistance to androgen/AR-targeted therapy

**DOI:** 10.1038/s41467-018-07872-w

**Published:** 2019-01-08

**Authors:** Hao Geng, Changhui Xue, Janet Mendonca, Xiao-Xin Sun, Qiong Liu, Patrick N. Reardon, Yingxiao Chen, Kendrick Qian, Vivian Hua, Alice Chen, Freddy Pan, Julia Yuan, Sang Dang, Tomasz M. Beer, Mu-Shui Dai, Sushant K. Kachhap, David Z. Qian

**Affiliations:** 1 OHSU Knight Cancer Institute, Prostate Cancer Program, Oregon Health & Science University, 3181 SW Sam Jackson Park Road, Portland, OR 97239 USA; 2 Johns Hopkins Kimmel Cancer Center, 401 N Broadway, Baltimore, MD 21287 USA; 3 Department of Medical Genetics, Oregon Health & Science University, 3181 SW Sam Jackson Park Road, Portland, OR 97239 USA; 4 NMR Core facility, Oregon State University, Corvallis, OR 97331 USA; 5 Division of Hematology & Medical Oncology, Oregon Health & Science University, 3181 SW Sam Jackson Park Road, Portland, OR 97239 USA

Correction to: *Nature Communications* (2018) 9:4972; 10.1038/s41467-018-07411-7; published online 26 November 2018

The original version of this Article contained errors in Fig. 7. In panels e and f, the graph titles incorrectly read ‘LNCaP-AdtNs’ and ‘LAPC4-AdtNs’, respectively. These errors have now been corrected in both the PDF and HTML versions of the Article.

