# Supplementary material for: Interplay between hypoxia and androgen controls a metabolic switch conferring resistance to androgen/AR-targeted therapy
Source: Nat Commun. 2018 Nov 26;9:4972. doi: 10.1038/s41467-018-07411-7 (PMC6255907; doi:10.1038/s41467-018-07411-7)
Supplement: Supplementary file 1 — Supplementary Information [file 41467_2018_7411_MOESM1_ESM.docx]

**Supplementary Information for Interplay between hypoxia and androgen controls a metabolic switch conferring resistance to androgen/AR-targeted therapy (Geng et al)**

**Supplementary Fig 1**

Venn diagram showing keyword search on PubMed: the search for cancer and hypoxia led to 24,295 hits; the search for cancer and androgen lead to 41,157 hits; the search for cancer, hypoxia, and androgen only led to 209 hits.

**
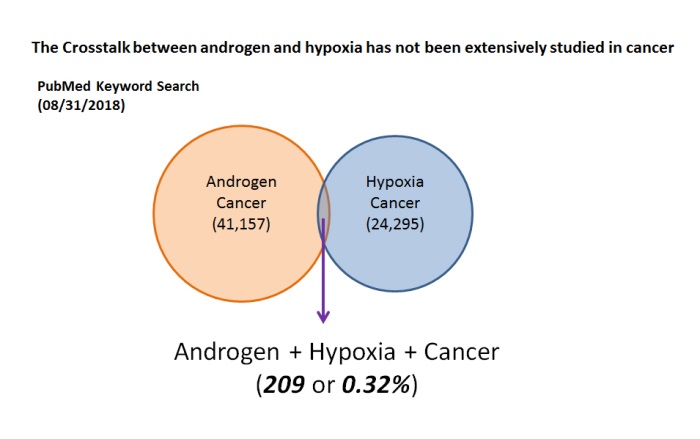
**

**Supplementary Fig 2**

Efficacy of AR-siRNA, (a) Representative western blots testing the efficacy of AR-siRNA, the siAR #1 (Sigma) was used throughout the study. (b) AR was inhibited by siAR #1 in normoxia and hypoxia.


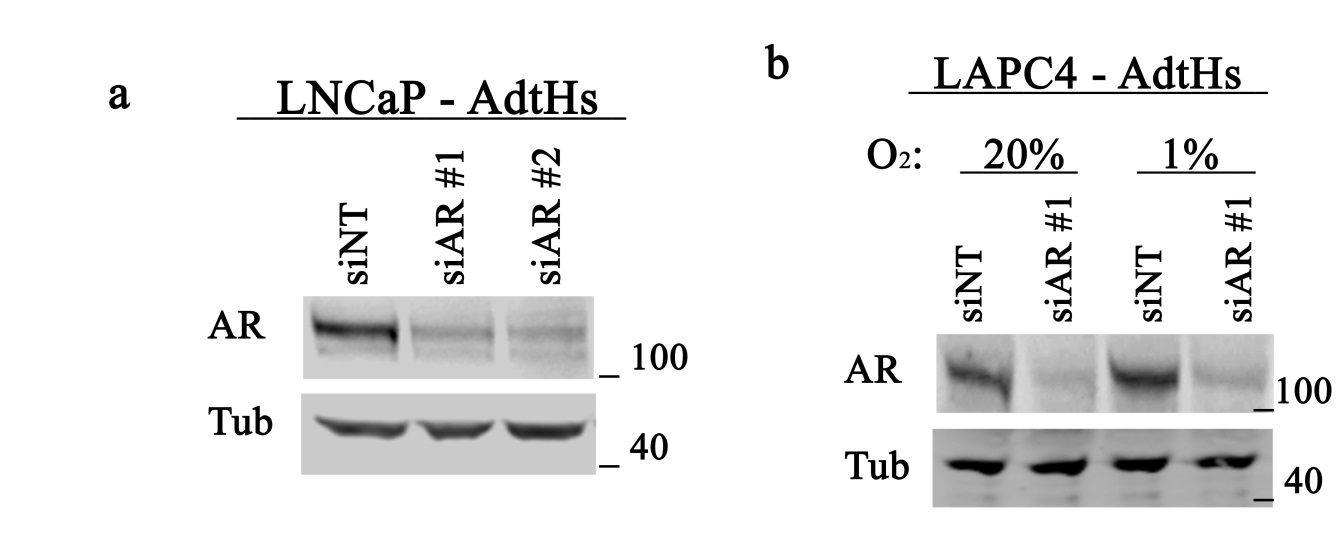


**Supplementary Fig 3**

Androgen attenuates subsets of hypoxia-response cancer hallmark pathways and genes. (a) The cancer hallmark pathways that were enriched by hypoxia in the absence (-) or presence (+) of androgen R1881. * FDR-q < 0.25, hypoxic effect in castration vs. in androgen, GSEA. The enrichment score was derived by hypoxia without androgen vs. normoxia without androgen, or hypoxia with androgen vs. normoxia with androgen. The FDR-q was derived by hypoxia without androgen vs. hypoxia with androgen. (b) The cancer hallmark pathways that were enriched by ADT in hypoxia or ADT in normoxia. * FDR-q < 0.25, ADT in hypoxia vs. ADT in normoxia, GSEA. The enrichment score was derived by ADT in normoxia vs. androgen in normoxia, or ADT in hypoxia vs. androgen in hypoxia. (c – e) Pathways and corresponding genes were upregulated by ADT in hypoxia compared to ADT in normoxia. The androgen attenuated hypoxia response genes identified by FDR-t-test in Fig 2d are in bold. (f) List of androgen-attenuated hypoxia response genes. (g) List of androgen-attenuated hypoxia response genes confirmed with qRT-PCR. A negative NES means enrichment due to the down regulation.


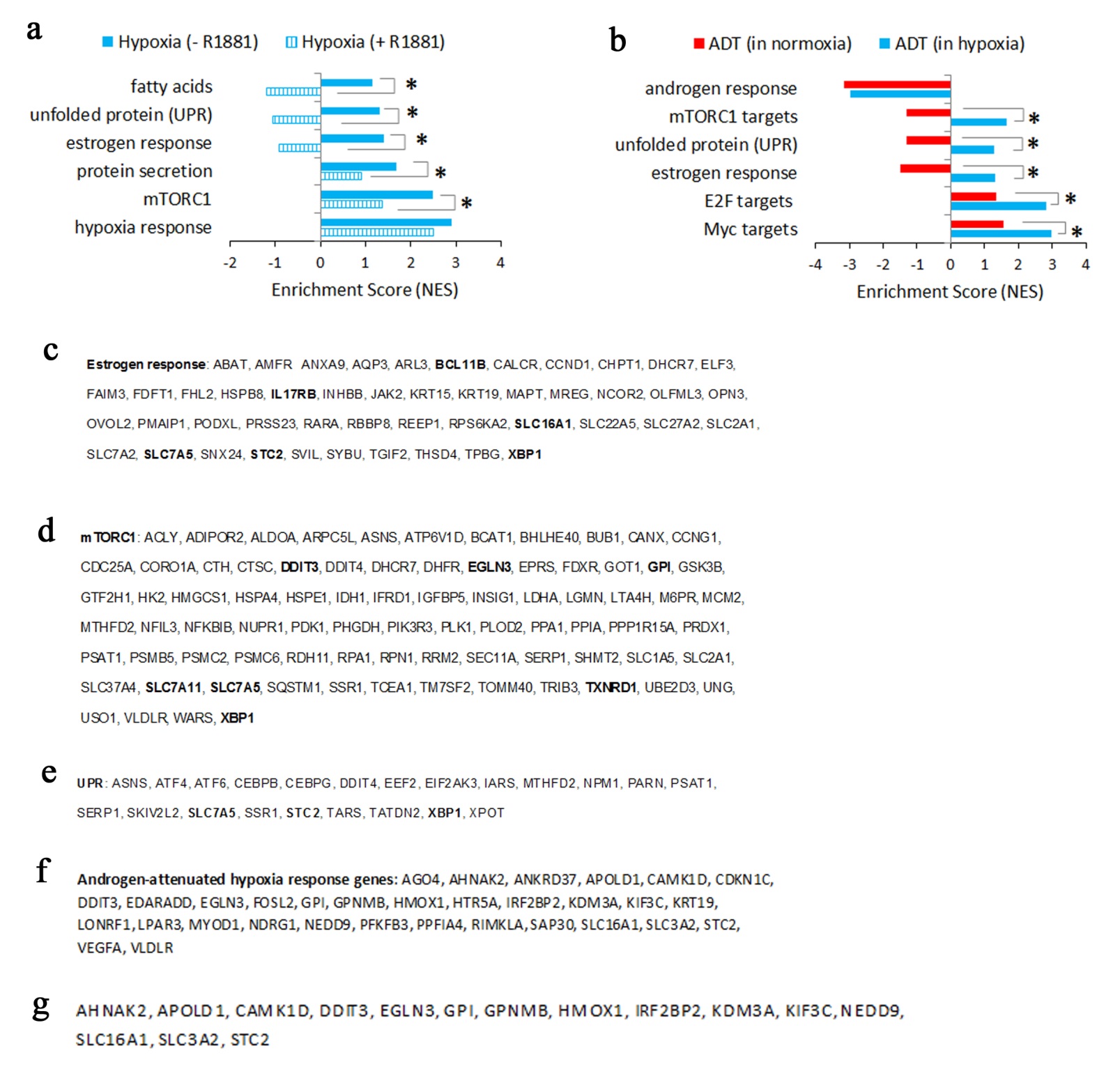


**Supplementary Fig 4**

GPI overexpression is implicated in clinical prostate cancer. (a) Kaplan-Meier analysis of disease free survival in relationship to GPI overexpression in prostate cancer TCGA dataset n = 491. (b) The % of patients with GPI overexpression determined by cBioPortal in six prostate cancer clinical datasets, see the Experimental Procedure section for detail, NEPC = Neuroendocrine prostate cancer.


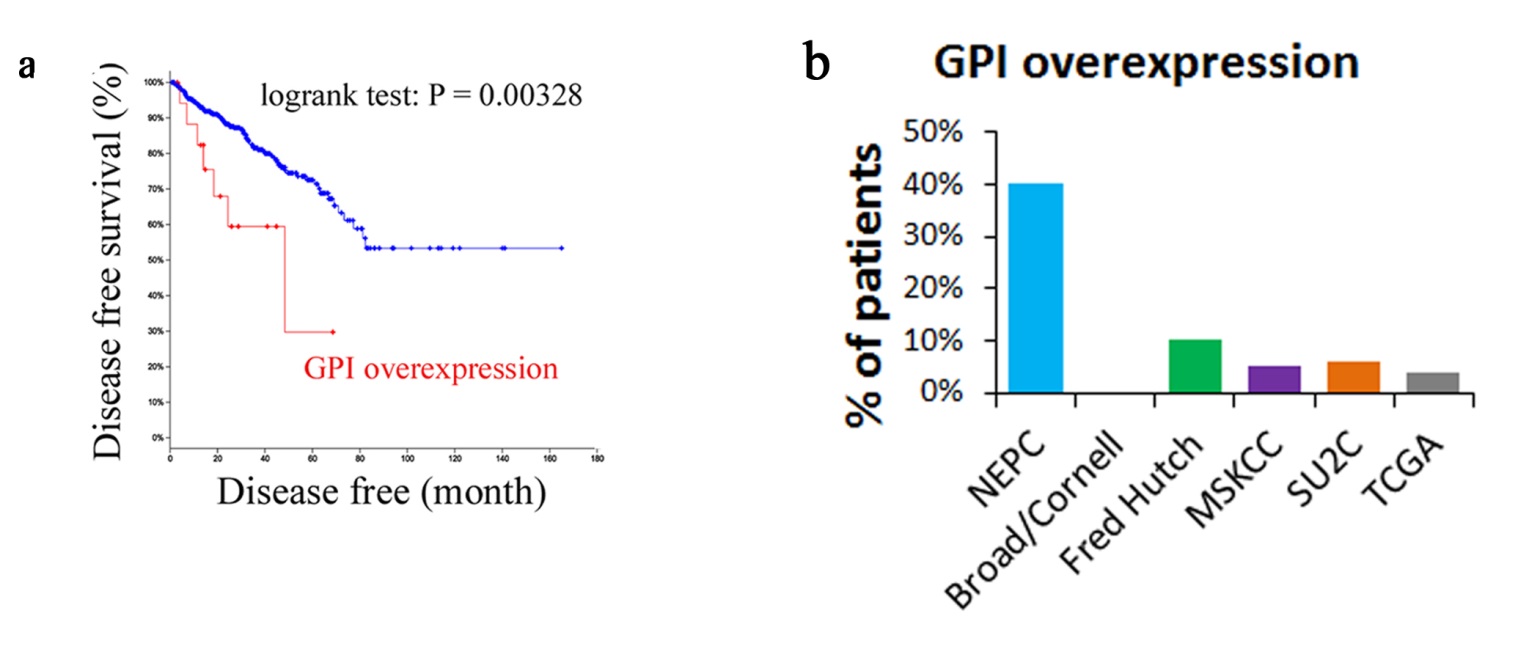


**Supplementary Fig 5**

AR attenuates hypoxia-induced GPI upregulation. **(a)** The change of GPI mRNA in LAPC4 cells in response to R1881 (1 nM) or enzalutamide (10 µM) in hypoxia. All cells were treated with the indicated conditions for 48 hours; basal was CSS media without androgen. GPI mRNA was determined by qRT-PCR. All values were relative to the basal-solvent condition, mean + s.d. n = 3, *, ^#^P < 0.05, **, ***P < 0.01, t-test and/or repeated measures ANOVA. **(b)** Relative GPI enzymatic activities in LAPC4 cells that were cultured with CSS + 1 nM R1881, and treated by enzalutamide (Enza) or solvent control (R1881) in normoxia (N) or hypoxia (H) for 48 hours, mean + s.d. n = 3, *P < 0.05, t-test. **(c)** GPI mRNA levels in LAPC4-AdtHs cells in the presence of siRNA silencing HIF1α, HIF2α, AR, or non-targeting control (siNT). Cells were transfected with siRNA, and cultured in the indicated conditions for 72 hours. GPI mRNA is relative to basal, mean + s.d., n = 3, **P < 0.01, t-test and/or repeated measures ANOVA. **(d)** GPI mRNA levels in DU145+Ev (Ev) or DU145+AR (AR) cells. Cells were treated by negative control (Ctl), 1 nM R1881, or siAR for 48 hours in normoxia or hypoxia. GPI mRNA is relative to Ctl-Ev at 20% O_2_, mean + s.d., n = 3, *P < 0.05, **P < 0.01, t-test.


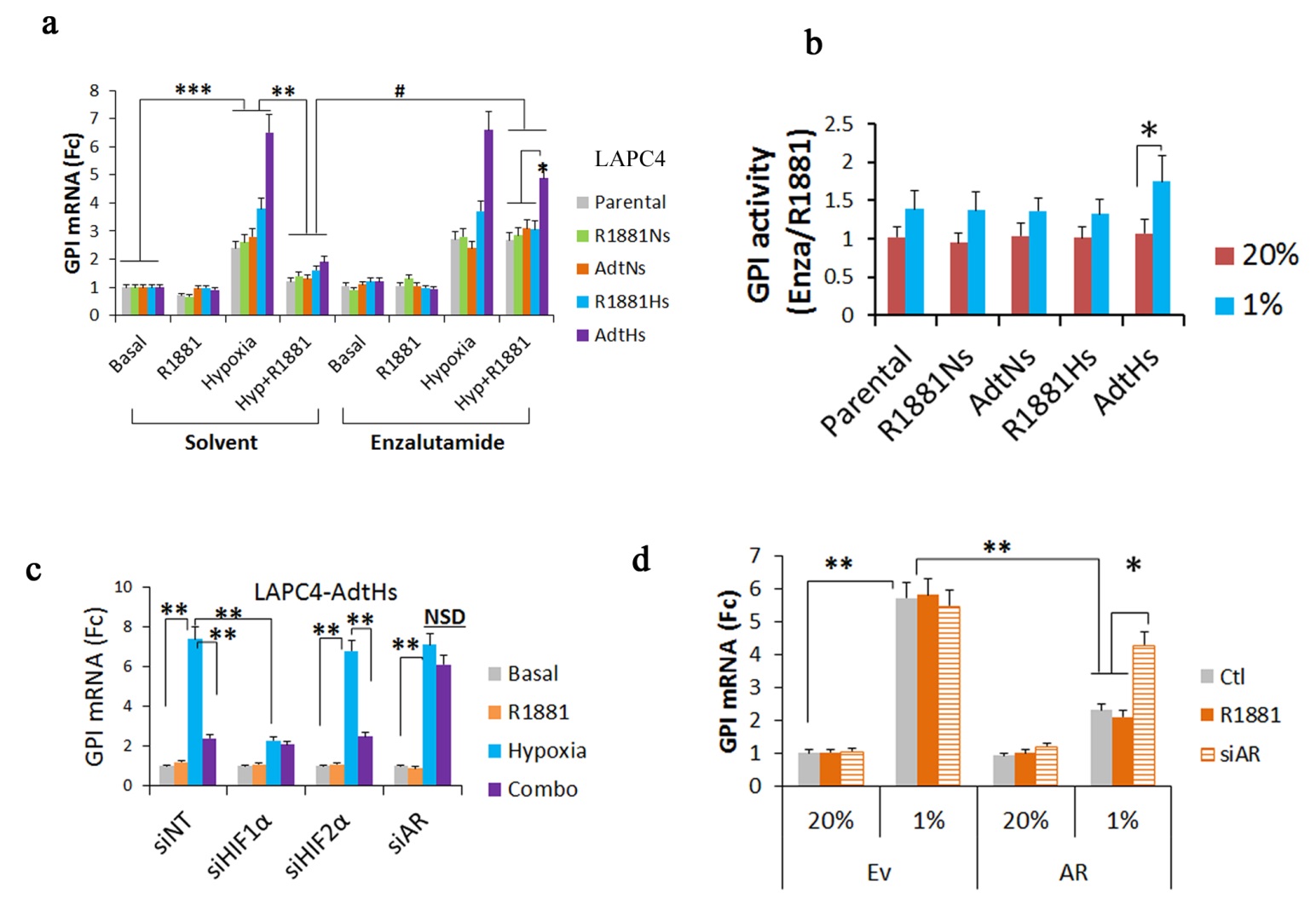


**Supplementary Fig 6**

The promoter sequences in the luciferase reporter genes for GPI and ENO1. The HRE is underlined in blue. AREhs is underlined in red. The blue sequence in ENO1 promoter was functionally characterized as hypoxia response and HIF1 binding region (JBC 271: pp. 32529, 1996).

**GPI promoter sequence in the promoter-driven luciferase construct from SwitchGear Genomics**

5’-CCCAAAGTGATGAGATTACAGGCGTGAGCCACCGCGCCCGGCCTAGTCACTTTTTTCTTA

ATTGCAACCCCGATTCTCAGCACCCTTTTCATCTTGGTTTTCCTCAATAGCCCTTACCAC

CAGCAGACACACATCATCTGTTGTACTTGCTTATTTGGCACATATGTATCCACAGCGCCT

AGAACACTGCCTGTAACGTGGAAGGTGTTCGATCTATAGAGTTTTGTCGAATGAATGAAT

GAAGCCGACTAGTGCACAGGGAGTGCAGCGGCGCGATGGTAGCTCTCTGCAGCCTCCAAC

ACCTGGGCTCCAGTGATCCCCGGGCTCTGCCCACCCTCCCCACTGCCACTTCCGGGCAGA

GGCCAGCAAAGCGGCGGCGCAAGAGGTAGGGAGAGAGGAGCTGAGGCCCCAGATCAGCGG

CCGCGGGCAAGGTCGCTCAGCGGGCACCCGGCCTGGGTATCGGGGCGCGGGTCGGGGGCG

GGGCCGGGGCTCAGGGGTGGGGCCGGGCCGGGCCGGGCCGGGCGCCTGCGCCATAAAGGC

CGCCGCGCGCCCACGCGCCTCGCTTGCTGCGCGCTGCCGGCGCTCCTTCCTCCTCGGCTC

GCGTCTCACTCAGTGTACCTTCTAGTCCCGCCATGGCCGCTCTCACCCGGGACCCCCAGT

TCCAGAAGCTGCAGCAATGGTACCGCGAGCACCGCTCCGAGCTGAACCTGCGCCGCCTCT

TCGATGCCAACAAGGAC-3’

**ENO1 promoter sequence in promoter-driven luciferase construct from SwitchGear Genomics**

5’-GGTCTGTGTGAAATTCCAGTTCCAAATAATTCACATGAATGGTTCACAAGAATCCAGGTA
GGTTTGATCTAGTTCCCTAGATGGGAATGAGAGGGATGTAAAATTTGAGAGGCAACATGA
CATGGACTAGAGAGAGAATGAATTAACACTGGGGAAGCACGTGGGGGCGCGCCGGACACC
TAGAAGGCACTCAAAAAGTATGAGGTAGAATTAAAGGAAAGTGAACCTTCCTTTGGCTGT
CTCGGCCTGATTCTCACCGCAGCAAGGAGAAAGGGACAGGGTCACCTTGGCGGGTGCCAG
GCGGAGCCCGGCCAGACGGGCGCGGCTGGAGGGCAGCGCGCAGGCGCAGGCGGCGCACGT
GGCCCCGGACACGCAGCGCTGCGGCGGGGCCAGCGCCCCAACCCCGGAGTGGGGCCGAGG
CGCGCAGCCTCTCAACGACTCGACGCCAACGGGGTCTCTCGGATCGCGGCGCGAGGCAGG
AGGGGCGGGGCAGGAGGGCCGCGGCAGGCCCGGGCCGCCAAAGTTGTCAGCAAGGTCGAG
GGCCGGACGTGGGGCCCCAGAGCGACGCTGAGTGCGTGCGGGACTCGGAGTACGTGACGG
AGCCCCGAGCTCTCATGCCCGCCACGCCGCCCCGGGCCATCCCCCGGAGCCCCGGCTCCG
CACACCCCAGTTCGGCTCACCGGTCCTATCTGGGGCCAGAGTTTCGCCCGCACCACTACA
GGGCCGCTGGGGAGTCGGGGCCCCCCAGATCTGCCCGCCTCAAGTCCGCGGGACGTCACC
CCCCTTTCCACGCTACTGCAGCCGTCGCAGTCCCACCCCTTTCCGGGAGGTGAGGGAATG
AGTGACGGCTCTCCCGACGAATGGCGAGGCGGAGCTGAGGGGGCGTGCCCCGGAGGCGGG
AAGTGGGTGGGGCTCGCCTTAGCTAGGCAGGAAGTCGGCGCGGGCGGCGCGGACAGTATC
TGTGGGTACCCGGAGCACGGAGATCTCGCCGGCTTTACGTTCACCTCGGTGTCTGCAGCA
CCCTCCGCTTCCTCTCCTAGGCGACGAGACCCAGTGGCTAGGTAAT-3’

**Supplementary Fig 7**

The Efficacy of GPI siRNA and overexpression plasmid. **(a)** Representative western blots testing the efficacy of GPI siRNA. LNCaP–AdtHs cells were transfected with 3 different cocktails of GPI siRNA constructs, and then cultured in hypoxia with CSS media without R1881 for 48 hours. The siGPI #1 (Sigma) was used throughout the study. **(b)** LAPC4 cells were transfected with plasmids coding for Flag-tagged GPI (0.2, 0.5 or 1.0 µg per transfection) or empty vector (Ev). The GPI antibodies detected both endogenous (endo) and overexpressed (OE) Flag-GPI. **(c)** LAPC4 cells were transfected with siNT (Ctl), siGPI, or siGPI + GPI-overexpression plasmid. Then cells were cultured in ADT or 1 nM R1881 in normoxia or hypoxia for 48 hours. GPI activities relative to Ctl with R1881/20% O_2_ were determined with a kit from BioVision, mean + s.d. n = 3. * P < 0.05, # P < 0.01, t-test.


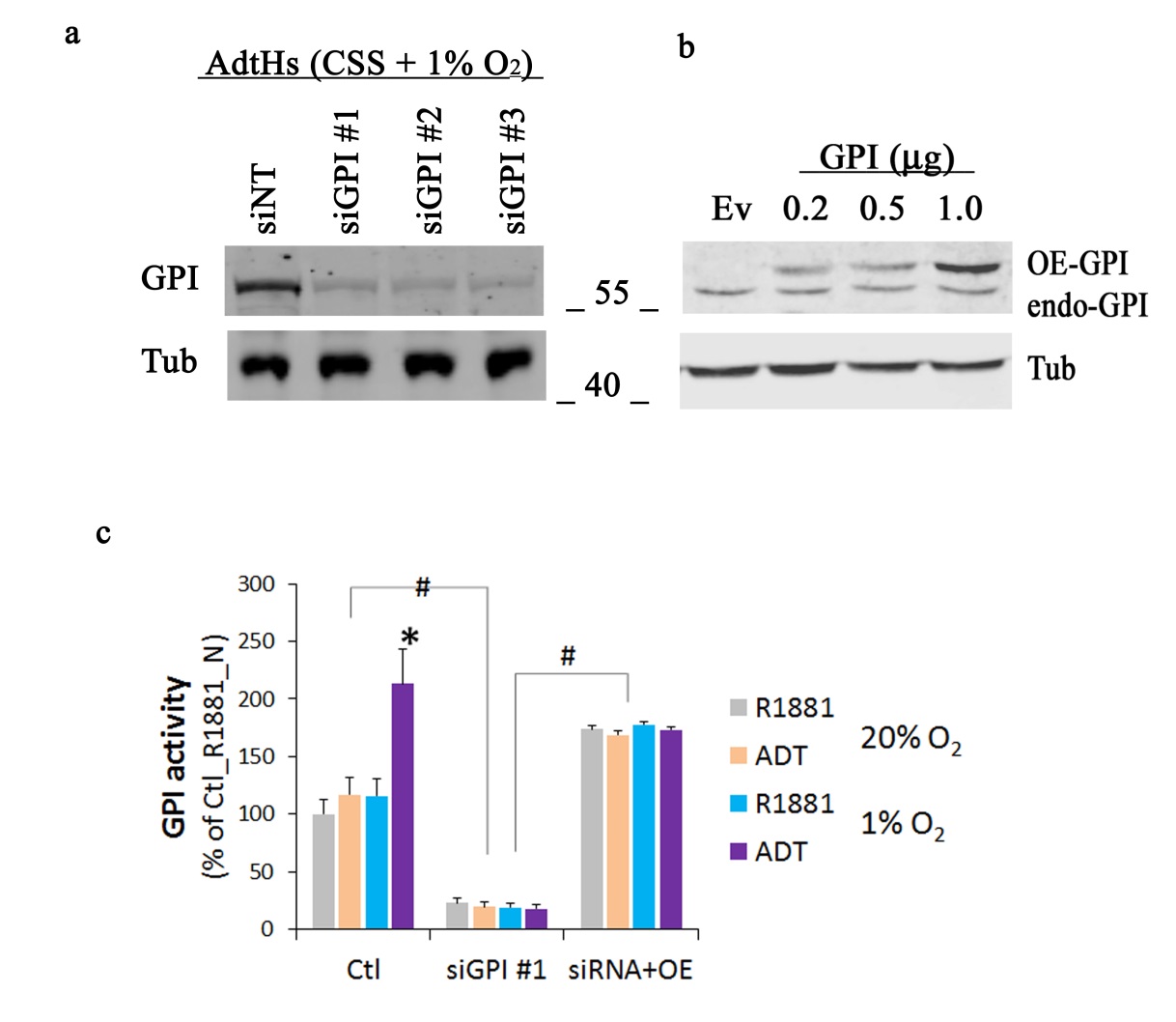


**Supplementary Fig 8**

AR overexpression alters glucose metabolism in hypoxia. **(a – b)** Levels of PPP metabolite 6-PGA (a) and glycolysis metabolite F6P (b) in DU145+Ev and DU145+AR cells were determined in the presence of 2DG or solvent control in 20% or 1% O_2_. All cells were treated by GPI metabolic inhibitor 2DG (10 mM) or solvent control (solv) in normoxia or hypoxia for 48 hours. All values were relative to solvent-treated Ev cells in normoxia, mean + s.d., n = 3, *,^#^ P < 0.05, NSD, t-test.


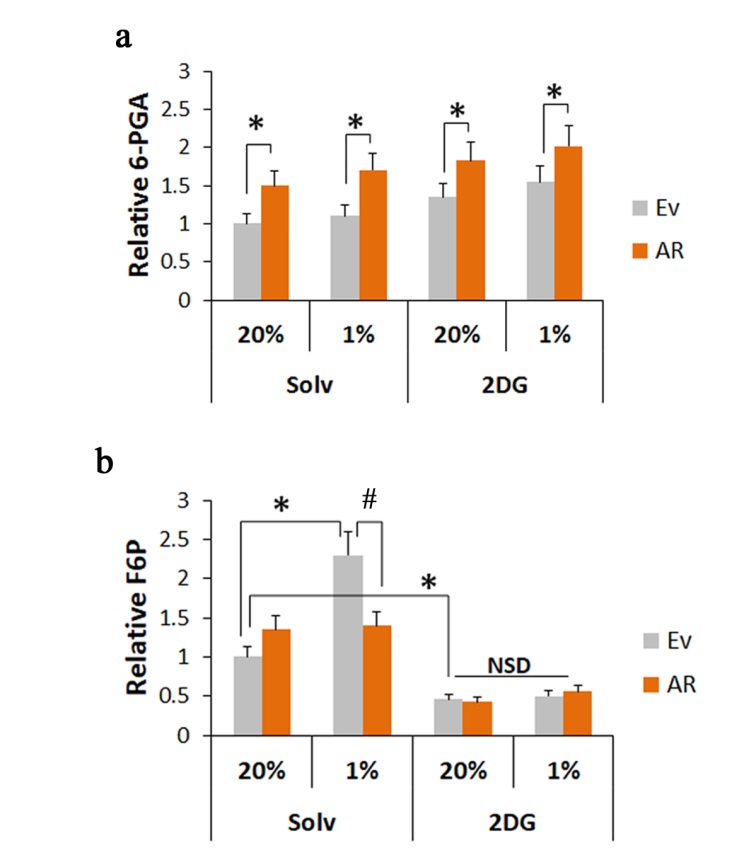


**Supplementary Fig 9**

Does-dependent viability/growth inhibition by enzalutamide to AdtNs cells in normoxia or hypoxia with or without GPI-siRNA. LNCaP and LAPC4 AdtNs cells, cultured in CSS media + 1 nM R1881, were transfected with siRNA (siNT or siGPI), and treated with increasing doses of enzalutamide in normoxia or hypoxia for 96 hours. Viable cells were determined by SYTO-60, mean + s.d. relative to 0 nM enzalutamide, n = 3.


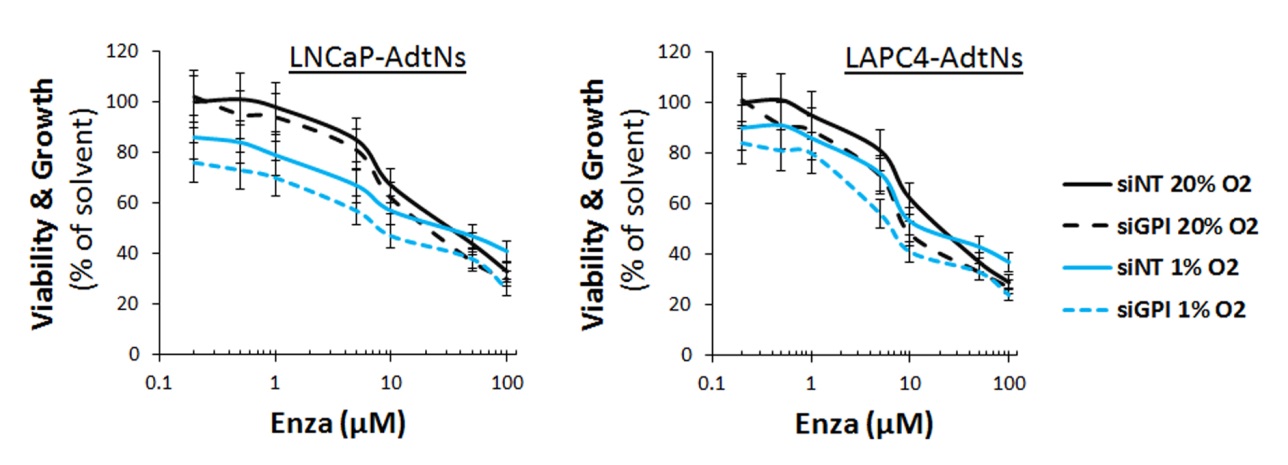


**Supplementary Fig 10**

Growth/viability of LAPC4-AdtHs cells in normoxia or hypoxia, being treated by increasing doses of enzalutamide, 2DG, or the combination for 96 hours. Viable cells were determined by SYTO 60, mean + s.d. relative to drug-free condition, n = 3, **P< 0.01, t-test.


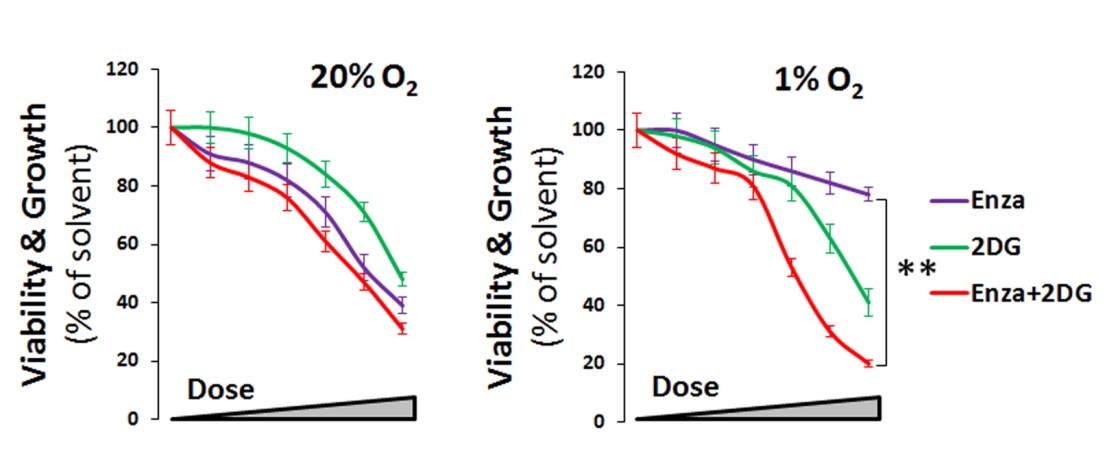


**Supplementary Fig 11**

GPI knockout by CRISPR/Cas9. **(a)** GPI in LNCaP-AdtHs cells was stably knocked out (KO) by CRISPR/Cas9. The resulted cells were cultured with androgen-free CSS media in normoxia (N) or hypoxia (H) for 24 hours, and GPI, HIF1α, and tubulin (Tub) were determined by western blots. **(b – d)** The Ctl and GPI-KO cells in (a) were cultured with 1 nM R1881 and treated with solvent or 10 µM enzalutamide in normoxia or hypoxia for 48 hours. GPI activity (b), PPP-metabolite 6-PGA (c), and glycolysis/GPI-metabolite F6P (d) were determined with kits from BioVision. **(e)** The dose-dependent survival/growth inhibitory effect of enzalutamide in Ctl or GPI-KO cells. All values are mean + s.d., n = 3.


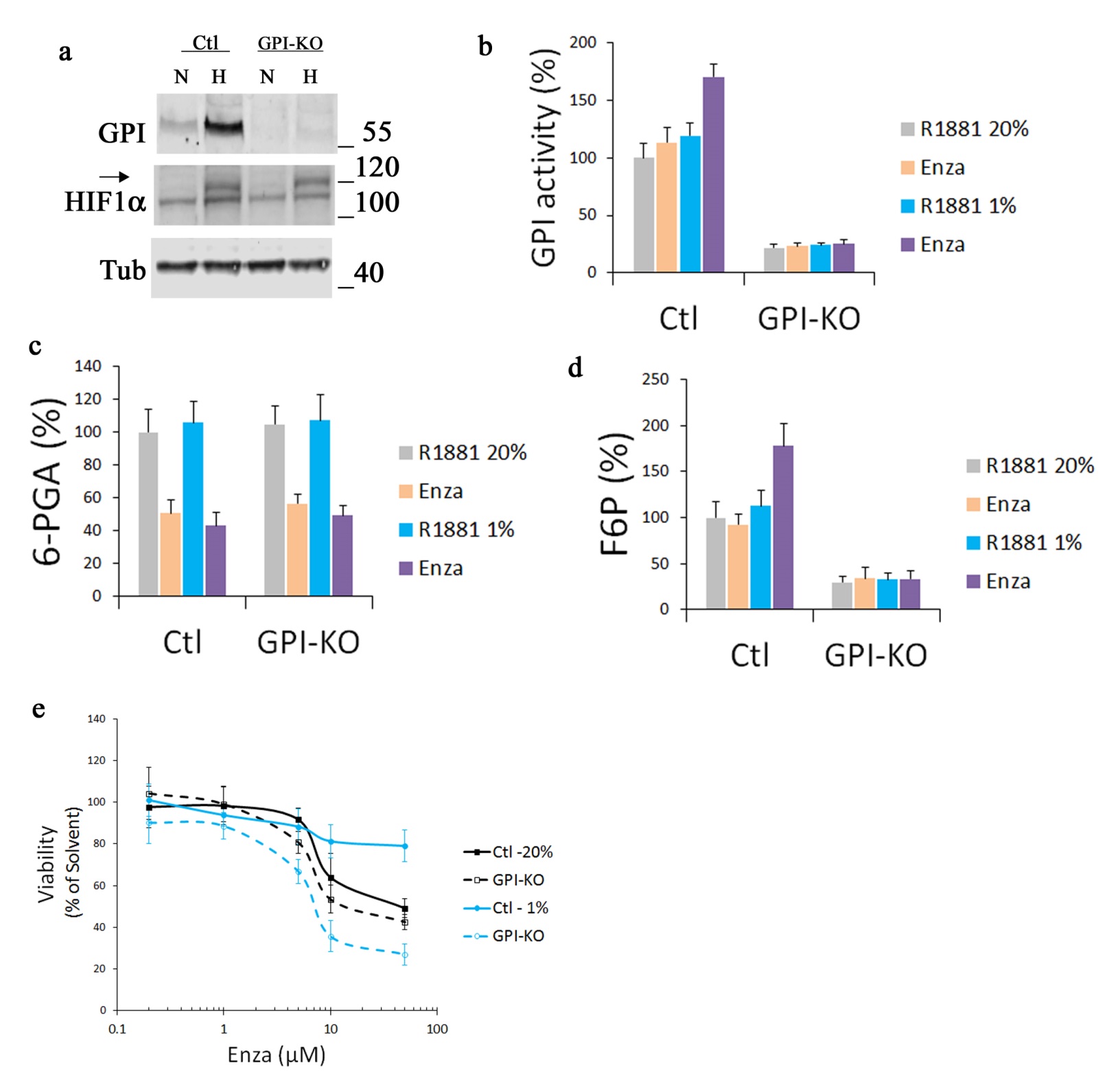


**Supplementary Fig 12**

Schematic demonstration of heterogenic resistant clones selected by the therapy. Each color represents a resistant clone due to an independent resistant mechanism, the color purple represents resistance due to conditional (hypoxic) androgen/AR-independence. It is also possible that multiple mechanisms exist in the same clone.


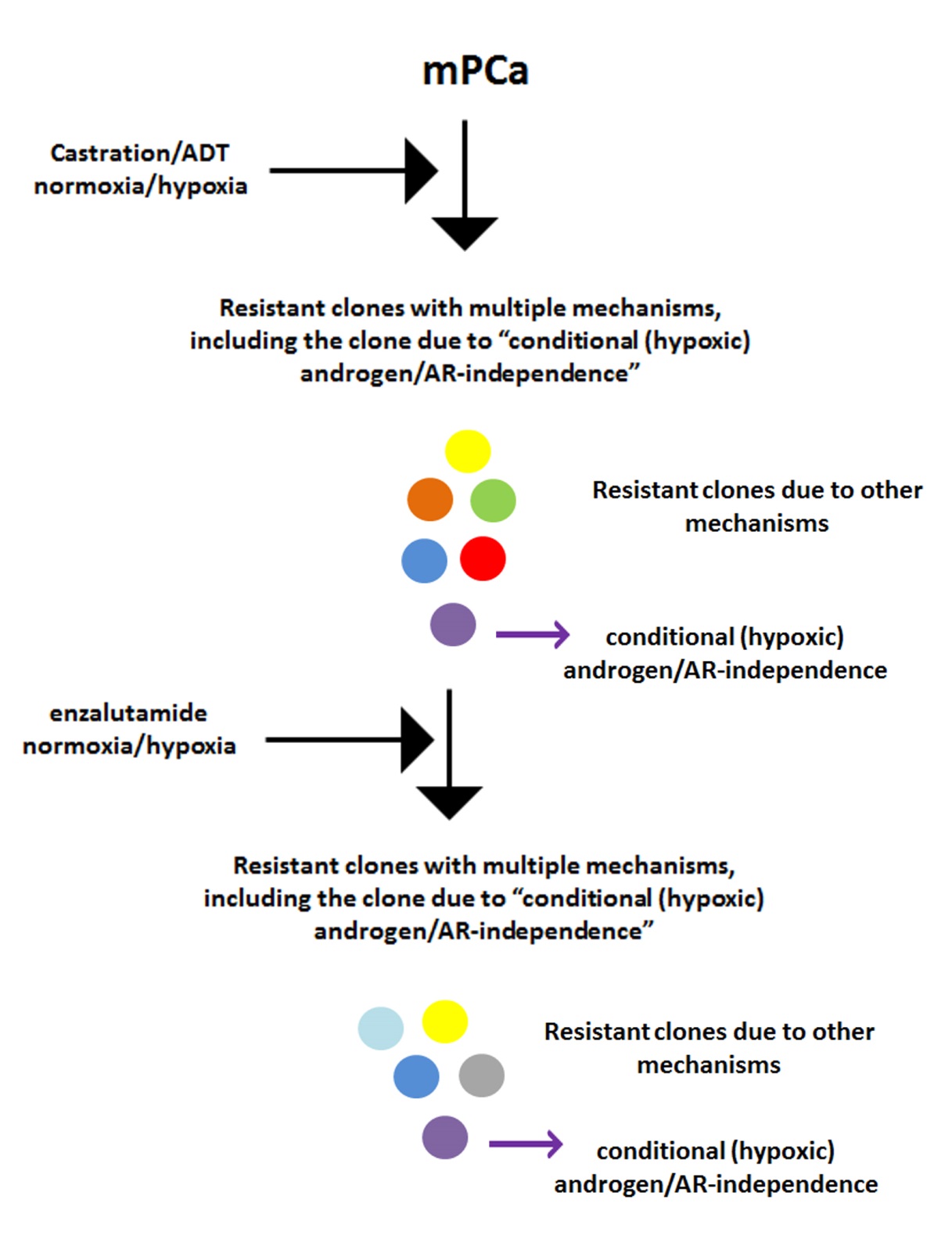


Supplementary Fig 13

**Western blot originals**

**
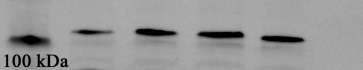
 Fig 3c_AR
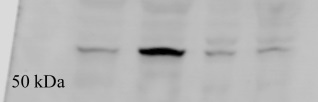
 Fig 3c_GPI**

**
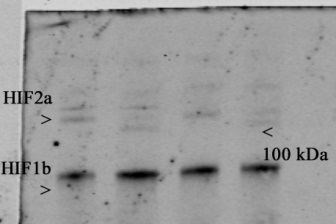
 Fig 3c_HIF2a and HIF1b
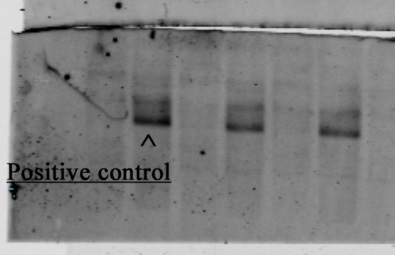
 Fig 3c_HIF1a**

**
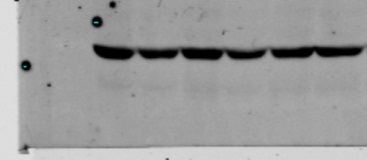
 Fig 3c_Tubulin**
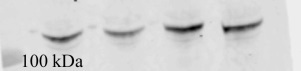
 **Fig 3d_AR**


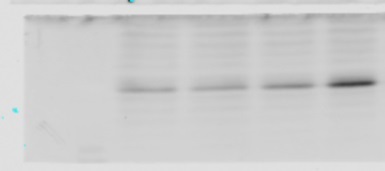
 Fig 3d_GPI
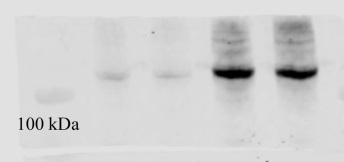
 Fig 3d_HIF1a


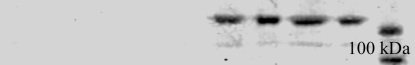
 Fig 3f_AR
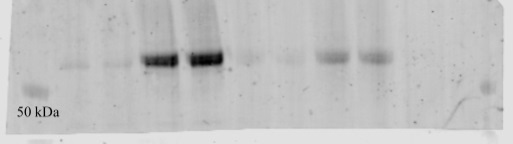
 Fig 3f_GPI


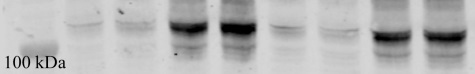
Fig 3f_HIF1
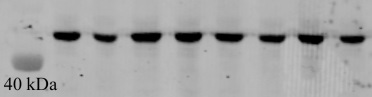
 Fig 3f_Tubulin


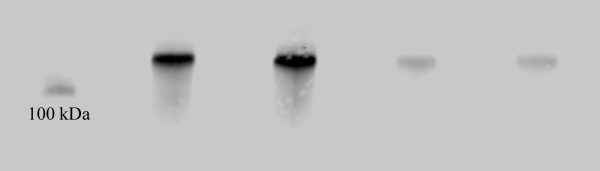
 Fig 3g_AR
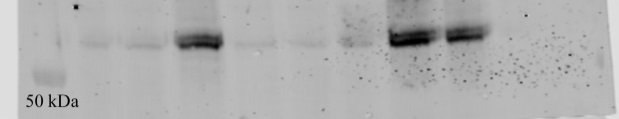
 Fig 3g_GPI


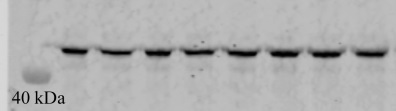
 Fig 3g_Tubulin

Supplementary Fig 14

**Primer sequences for qRT-PCR**

| Gene name | Accession # | Forward (5’-3’) | Reverse (5’-3’) |
| --- | --- | --- | --- |
| AHNAK2 | NM_138420 | GTGCAGAAACGGAAGATGACC | GCCTCAGTCGTGTATTCGTAGA |
| APOLD1 | NM_001130415 | CATACATGCAAAAACGGTGC | TCAGACATACTGCCCCATCA |
| CAMK1D | NM_020397 | TGCTGTGAAGTGTATCCCTAAGA | TCTCAGGACGGCTATCTCATTC |
| DDIT3 | NM_001195055 | GGAAACAGAGTGGTCATTCCC | CTGCTTGAGCCGTTCATTCTC |
| EGLN3 | NM_022073 | CTGGGCAAATACTACGTCAAGG | GACCATCACCGTTGGGGTT |
| GPNMB | NM_001005340 | CTTCTGCTTACATGAGGGAGC | GGCTGGTGAGTCACTGGTC |
| HMOX1 | NM_002133 | AAGACTGCGTTCCTGCTCAAC | AAAGCCCTACAGCAACTGTCG |
| IRF2BP2 | NM_001077397 | ACACCCATTTTGTGCAGTGC | ACTGGGACAATAGACCTCTCC |
| KDM3A | NM_001146688 | GTGCTCACGCTCGGAGAAA | GTGGGAAACAGCTCGAATGGT |
| KIF3C | NM_002254 | CACCTTTGACGCCGTGTATGA | CACCGTGCCATTGAAACCC |
| NEDD9 | NM_182966 | ATGGCAAGGGCCTTATATGACA | TTCTGCTCTATGACGGTCAGG |
| SLC16A1 | NM_001166496 | TCTGTGTCTATGCGGGATTCTT | TTGAGCCGACCTAAAAGTGGT |
| SLC2A3 | NM_006931 | GCTGGGCATCGTTGTTGGA | GCACTTTGTAGGATAGCAGGAAG |
| STC2 | NM_003714 | GGGTGTGGCGTGTTTGAATG | TTTCCAGCGTTGTGCAGAAAA |
| TMPRSS2 | NM_001135099 | GGACAGTGTGCACCTCAAAGAC | TCCCACGAGGAAGGTCCC |
| FKBP5 | NM_004117 | GCGGAGAGTGACGGAGTC | TGGGGCTTTCTTCATTGTTC |
| GPI | NM_000175 | AGGCTGCTGCCACATAAGGT | CCAAGGCTCCAAGCATGAAT |
| PSA | NM_001648 | TGTGTGCTGGACGCTGGA | CACTGCCCCATGACGTGAT |
| ENO1 | NM_001201483 | AGTCCCGGCGTTCAATGTC | GGCGGCTATGAGAGGTTTTCC |
| KLK2 | NM_001256080 | TCAGAGCCTGCCAAGATCAC | CACAAGTGTCTTTACCACCTGT |
